# Cancer Cell Metabolism

**DOI:** 10.3390/ijms23137210

**Published:** 2022-06-29

**Authors:** Eric K. Parkinson, Sebastian Haferkamp, Maria E. Mycielska

**Affiliations:** 1 Centre for Immunobiology and Regenerative Medicine, Queen Mary University of London, London E1 2AD, UK; 2 Department of Dermatology, University Hospital of Regensburg, 93053 Regensburg, Germany; 3 Department of Structural Biology, Institute of Biophysics and Physical Biochemistry, University of Regensburg, 93053 Regensburg, Germany

## 1. Introduction

Cancer metabolism has been of interest for decades; however, the recent development of sophisticated techniques such as metabolomics or lipidomics have significantly increased our understanding of processes taking place in tumour cells. Cancer cells not only need to have an active metabolism to produce enough energy and the building blocks necessary for increased proliferation but also to adjust to the changing extracellular conditions in the process of metastasis. The Warburg effect and increased glucose consumption are considered one of the hallmarks of cancer. Interestingly, however, the ability of cancer cells to use OXPHOS for energy synthesis has been recently recognised as playing an important role in the process of metastasis (reviewed by [1]). It can be therefore deduced that cancer cell metabolism must differ significantly from normal cells and be a potentially good target to treat this disease.

## 2. Contributions to the Research Topic

Warburg effect—aerobic glycolysis—is the best known and still not fully understood cancer phenomenon. Although ATP synthesis in mitochondria used by normal cells is more efficient and secures the appropriate redox balance control, cancer cells tend to increase their glucose consumption and disrupt redox regulation by favouring ATP synthesis through glycolysis. The advantages of using the Warburg effect such as the increased synthesis of nucleic acids, proteins and lipids is discussed in the review by Kubicka et al. [2]. Differences in the use of OXPHOS versus aerobic glycolysis have also been studied in the context of prognostic markers for urothelial bladder cancer [3]. To evaluate this possibility, a metabolomic study was performed on three cell lines of different grades. The obtained results showed that cells representing the highest grade rely mainly on aerobic glycolysis, while low grade cancer cells use OXPHOS as the predominant way of acquiring energy. Based on these results, the authors concluded that glycolytic activity correlates with a high risk of bladder cancer. The authors further suggested that the different metabolic states are linked to whether the cell lines possess TP53 mutations (glycolysis) or FGR3 mutations (OXPHOS). The authors acknowledge their data are preliminary in nature due to the small number of lines studied. However, their data may provoke more extensive studies on in vivo bladder cancer samples linking gene mutations to metabolic states.

Consistently, metabolic syndromes with high serum glucose levels have been positively correlated with an increased risk of colorectal cancer as reviewed by Kasprzak [4]. The author focused on the role of insulin-like growth factor 1 (IGF1) with the aim of drawing links between risk factors such as a high glucose diet, type 2 diabetes and in particular, the Warburg effect. The author also highlighted the current interest in metformin as an anti-cancer strategy as well as several other potential ways of targeting IGF1 signalling.

Recent studies revealed that although the Warburg effect is crucial in the disease progression, cancer cells need to be able to switch between aerobic glycolysis and OXPHOS, especially in the process of metastasis. Silencing of Fer kinase disabled the possibility of cancer cells to use OXPHOS and made them heavily dependent on glucose and, therefore, reduced their metabolic plasticity as shown by Mehazri et al. [5]. These authors investigated the role of Fer kinase on a non-small cell lung cancer line and a triple negative breast cancer line by knocking out the enzyme in both sets of cells using CRISPR-cas9 technology and testing the effects of glucose deficiency on mitochondrial function and NAD+ metabolism, both of which were impaired in the knockout cells. The cells show multiple defects in mitochondrial function coupled with increased rates of DNA damage and apoptosis. Most importantly, the authors demonstrated reduced tumour xenograft growth and the effect was potentiated by a low carbohydrate ketogenic diet. The data suggest that the Fer kinase could be an anti-cancer target especially if coupled with a low carbohydrate intake.

Citrate has been considered to be the central metabolite in cancer, giving rise to fatty acid synthesis, and therefore fast metabolism. Citrate can be either synthesised intracellularly in the forward or reverse Krebs cycle or can be imported from outside [6,7]. Consistently, cancer cells express a specific plasma membrane transporter, pmCiC, which facilitates citrate uptake. Although citrate is necessary for the proper functioning of cancer cells, its increased intracellular level can be detrimental to cancer metabolism [8]. Consequently, increasing citrate levels by forced uptake was discussed in the context of a novel anti-cancer therapy by Icard et al. [9].

The reduced use of mitochondria by cancer cells challenges the redox balance and has been studied extensively in this regard. NAD^+^ is part of the redox balance and is necessary to sustain glycolysis. Franceschini et al. [10] demonstrated a potential anticancer applicability of targeting nicotinamide phosphoribosyl transferase (NAPRT), the rate-limiting enzyme in the formation of the NAD^+^, in the treatment of osteosarcoma (OS). They used the NAPRT inhibitor FK866 to inhibit the enzyme in OS lines as NAPRT is often over-expressed in this type of cancer in vivo. NARPT mediates the alternative salvage pathway from nicotinic acid to sustain NAD+ levels. The authors tested several OS lines in vitro and showed that FK866 reduced OS cell line proliferation and differentiation. However, the results were variable and the authors suggest that only lines with low levels of NAPRT respond well to FK866 and that this may be due to the partial methylation of the NAPRT gene promoter. Based on this last observation, the authors suggested that NAPRT expression levels could be used to select OS patients for potential response to FK866.

Fast growth of primary tumours is often accompanied by low-oxygen conditions. In this context, Infantino et al. [11] discussed the cross-talk between hypoxia inducible factor 1 (HIF-1) and mitochondria in hypoxic cancer cells with HIF-1 as a potential target. In addition, hydrogen sulphide (H_2_S) producing enzymes are upregulated in different cancer types. H_2_S mediated signalling pathways have been reviewed as other metabolic targets in cancer therapy [12]. In particular, the role of H_2_S as a vasodilator would indicate a potential role in tumour angiogenesis and the maintenance of tumour blood supply. The following three enzymes: cystathionine-synthase cystathionine-lyase), and 3-mercaptopyruvate sulfotransferase, regulate the production of H_2_S and all three are upregulated in various cancers. However, at present, there are no drugs that specifically target these enzymes and there are many downstream effectors of H_2_S, so further investigation into the H_2_S mediated signalling is required.

Another approach in the search of new cancer treatments is drug repurposing as new drugs are expensive to produce and take through to phase 1 trials. Duarte et al. [13] showed the potential application of drugs used in the central nervous system (CNS) for use against colorectal and breast cancer. The authors found that the response to treatment depends on the origin of the cancer cell lines tested and that CNS drugs applied alone or in combination with chemotherapy might improve existing anti-cancer therapies. The role of the repurposed drugs in cancer cell metabolism is not presently known.

## 3. Conclusions

The collected manuscripts provide novel insights into many of the most important elements of cancer metabolism and suggest new options for anti-cancer treatments based on specific metabolic targets or by drug repurposing. Such studies are likely to be very important in the future for cancer types that have so far remained intractable for conventional or targeted therapies.
